# The heart of the futsal athletes: a comparison of heart structure among under-18, under-20 and adult elite players

**DOI:** 10.3389/fcvm.2025.1710254

**Published:** 2025-12-12

**Authors:** Luís Felipe Tubagi Polito, Yago de Moura Carneiro, Danilo de Figueiredo Biaggioni, Thomas Brolin Vieira Nascimento, Davi Ribeiro do Prado, Marcelo Villas Boas Junior

**Affiliations:** 1 Sport Club Corinthians Paulista—Health and Performance Center, São Paulo, SP, Brazil; 2 Center of Excellence in Exercise Physiology and Training—NEFET, São Paulo, SP, Brazil; 3 Sfera Futebol Clube—Health and Performance Center, Jarinu, SP, Brazil; 4 Department of Orthopedics and Traumatology, Hospital 9 de Julho, São Paulo, SP, Brazil; 5 Department of Physical Education, São Judas University Rua Taquari, São Paulo, SP, Brazil; 6 Study Group on Myology Applied to Exercise, Vila Monte Alegre, Ribeirão Preto, SP, Brazil; 7 University of São Paulo (USP) Ribeirão Preto School of Medicine (FMRP) Av. Bandeirantes, Ribeirão Preto, SP, Brazil

**Keywords:** futsal, cardiac remodeling, VO_2_max, left atrial volume, ventricular thickness, exercise physiology

## Abstract

**Introduction:**

This study examined cardiac adaptations in futsal athletes to determine how sport-specific training influences cardiac morphology and function across different competitive levels.

**Methods:**

Male athletes from under-18, under-20, and adult categories underwent electrocardiogram, transthoracic echocardiogram, and cardiopulmonary exercise testing. Measured parameters included VO₂max, ventricular and atrial dimensions, wall thickness, and cardiac mass index. Group differences were analyzed using one-way ANOVA with Tukey's *post-hoc* test (*p* < 0.05).

**Results:**

Under-20 athletes showed significantly higher VO₂max compared to adults (mean difference: +4.87 mL·kg^−1^·min^−1^; *p* = 0.014). Adult players exhibited greater interventricular septal (+0.68 mm; *p* = 0.048) and inferolateral left ventricular wall thickness (+0.75 mm; *p* = 0.016), alongside higher left atrial volume (+27.4 mL vs. U18; *p* < 0.001) and indexed left atrial volume (+14.6 mL/m² vs. U18; *p* < 0.001). Conversely, the right ventricular end-diastolic diameter was larger in under-18 athletes compared to under-20 (+10.9 mm; *p* < 0.001) and adult players (+14.3 mm; *p* < 0.001). Ejection fraction, left ventricular end-diastolic diameter, and ventricular mass index remained consistent among groups, confirming preserved systolic function across all athletes.

**Conclusion:**

Progressive futsal training promotes selective cardiac remodeling characterized by increased wall thickness and chamber dilation in adult athletes without compromising function. These adaptations reflect physiological remodeling associated with chronic high-intensity intermittent training, emphasizing the need for longitudinal monitoring to distinguish normal adaptation from early pathological changes.

## 1 Introduction

Among the various chronic adaptations promoted by high-level training, morphofunctional changes in the cardiac muscle stand out, a phenomenon often referred to as the athlete's heart (AH) (1, 2). It is well established that regular exercise training is associated with structural and functional modifications of the heart, including increased ventricular wall thickness, enlargement of the left (LV) and right (RV) ventricular cavities, atrial dilation, and functional changes such as reduced resting ejection fraction when compared to non-athletes (3, 4). Among the various chronic adaptations promoted by high-level training, morphofunctional changes in the cardiac muscle stand out, a phenomenon often referred to as the athlete's heart (AH) (1, 2). It is well established that regular exercise training is associated with structural and functional modifications of the heart, including increased ventricular wall thickness, enlargement of the left (LV) and right (RV) ventricular cavities, atrial dilation, and functional changes such as reduced resting ejection fraction when compared to non-athletes (3, 4). These cardiovascular adaptations are closely linked to improved aerobic fitness and cardiorespiratory efficiency, which can be objectively assessed through the Cardiopulmonary Exercise Test (CPET), an established method for evaluating maximal oxygen uptake and the integrative response of the cardiovascular and respiratory systems to exercise (5). Such adaptations represent a physiological response to repeated hemodynamic overload and could be reversible in the absence of training stimulus (6).

Exercise-induced adaptations are heterogeneous and may vary significantly according to sex, age, ethnicity, training status, and type of exercise performed (7–10). The process of cardiac remodeling involves changes in size, geometry, mass, and chamber function, allowing the heart to adapt to the magnitude of the hemodynamic stimulus, which is determined by the intensity, duration, and nature of exercise (6). Prolonged aerobic exercise increases preload, promoting eccentric LV hypertrophy, while isometric or resistance exercises may generate significant increases in afterload, inducing concentric hypertrophy patterns (1, 7).

The type of physical training is an important determinant of cardiac adaptation. Morganroth's classical hypothesis suggested that endurance athletes would develop eccentric LV hypertrophy, whereas strength athletes would present concentric hypertrophy (1). However, subsequent studies have questioned the generalization of this hypothesis, highlighting methodological limitations of cross-sectional studies and the need for longitudinal analyses to fully understand training-specific adaptations (11). Recent evidence indicates that although endurance training promotes an increase in end-diastolic volume and eccentric hypertrophy, the magnitude and pattern of adaptation are highly individualized, influenced by genetic factors, age, sex, and the onset of sports practice (3, 4, 12).

Futsal, as a mixed-modality sport, imposes substantial cardiovascular and physical demands, combining aerobic and anaerobic efforts in intermittent high-intensity patterns (13, 14). Players frequently alternate between walking, jogging, sprinting, and rapid changes of direction, generating both volume and pressure overload on the left ventricle. This results in an intermediate pattern of ventricular hypertrophy, positioned between the classic endurance and strength models, with proportional increases in intracavitary diameters and wall thickness (15). Physiologically, futsal differs from more conventional sports such as field soccer by maintaining high-intensity effort throughout most of the match. Futsal players demonstrate an average heart rate of 177 bpm and a VO_2_max of 63.4 mL·kg^−1^·min^−1^, sustaining approximately 95% of playing time near maximal oxygen consumption, whereas field soccer players exhibit lower values (167 bpm, VO₂max 52.5 mL·kg^−1^·min^−1^, and 76% of time at high intensity) (14). Moreover, both U20 and adult athletes perform repeated short sprints lasting 5–6 s with high mean anaerobic power (≈470–500 W) and low fatigue indices, highlighting the need for rapid recovery during matches (16–18).

Understanding cardiac adaptations in futsal athletes is crucial not only for optimizing sports performance but also for monitoring cardiovascular health. Detailed investigation of athlete's heart in mixed sports contributes to a broader understanding of exercise physiology and the prevention of cardiovascular pathologies in athletic populations. In this context, the present study aimed to examine possible structural cardiac alterations resulting from training in athletes from different categories of Brazilian elite futsal, providing insights into the physiological complexity of the sport and the specific cardiovascular demands placed on its athletes.

## 2 Materials and methods

### 2.1 Study group

A total of 58 athletes participated in the study, distributed into the following categories: under-18 (*n* = 24; mean age 17.3 ± 0.6 years; weight 78.5 ± 9.7 kg; height 1.73 ± 0.6 m), under-20 (*n* = 16; mean age 19.1 ± 0.7 years; weight 70.8 ± 8.8 kg; height 1.74 ± 0.7 m), and adults (*n* = 18; mean age 24.2 ± 8.8 years; weight 74.2 ± 8.5 kg; height 1.74 ± 0.4 m). All athletes belonged to a national elite futsal team. A convenience sample was selected, including athletes with a minimum of seven years of systematic practice in the modality and who were part of the official roster for the professional championship season. Exclusion criteria included athletes presenting any cardiometabolic comorbidity that could impair professional sports participation, as well as those who were smokers or using medications known to alter cardiac structure or function, such as beta-blockers.

The present study investigated possible cardiac alterations in professional futsal athletes through evaluations with complementary tests, including electrocardiogram (ECG), transthoracic echocardiogram (TTE), and cardiopulmonary exercise testing (CPET). The examinations were analyzed by cardiologists who followed national and international guidelines and recommendations for athlete assessment.

### 2.2 Statistical analyses

Data were initially presented using descriptive statistics, with means and standard deviations. To verify differences among athlete categories (under-18, under-20, and professional), a one-way analysis of variance (ANOVA) was applied. When a significant effect was identified, Tukey's *post hoc* test was performed for multiple group comparisons. All statistical analyses were conducted using SPSS, version 30.0.0 (IBM Corp., Armonk, NY, USA), while graphs were created using GraphPad Prism (GraphPad Software, San Diego, CA, USA). The level of significance was set at *p* < 0.05.

### 2.3 Electrocardiogram (ECG)

The 12-lead resting electrocardiogram was performed on all athletes in the supine position after a minimum rest period of 5 min, with recordings obtained at a speed of 25 mm/s. Interpretation followed the international recommendations for ECG interpretation in athletes (19). These recommendations, also known as the Seattle Criteria, aim to differentiate physiological adaptations in athletes from changes suggestive from ECG findings which might have a pathological underlying cause, which served as exclusion criteria in the present study.

### 2.4 Echocardiogram

The transthoracic echocardiogram was performed using a Philips Affiniti 70 ultrasound system equipped with an S5-1 phased-array transducer (1–5 MHz), following the recommendations of the American Society of Echocardiography (20). Left ventricular ejection fraction (LVEF), left ventricular mass index (LVMI), left ventricular end-diastolic diameter (LVEDd), and interventricular septum (IVS) thickness were obtained in M-mode from the parasternal long-axis window, with the transducer positioned perpendicular to the left ventricular axis at the level of the papillary muscles. LVEF was calculated using the Teichholz method (21), based on end-diastolic and end-systolic diameters of the left ventricle. LVMI was calculated using the Devereux formula, corrected for body surface area (22). LVEDd and IVS measurements were taken at end-diastole, with LVEDd defined as the distance between the septal wall and the posterior wall of the left ventricle, and IVS as the total thickness of the interventricular septum.

To ensure a fair comparison among athletes with different body sizes, the absolute values of the variables obtained from the examinations were indexed to body surface area. This procedure, which involves dividing each absolute measurement by the body surface area (BSA) of each individual, allows the results to be expressed as relative values. Such adjustment is essential, as it controls for the influence of body size on cardiac and physiological variables, enabling a more accurate interpretation of training-induced adaptations and reducing biases associated with differences in body structure.

Linear measurements were obtained using M-mode when proper parasternal long-axis alignment was ensured, as this technique provides high temporal resolution and reproducibility in serial evaluations.

### 2.5 Cardiopulmonary exercise test

The cardiopulmonary exercise test (CPET) was performed on a treadmill (INBRAMED ATL) using an incremental ramp protocol until the athlete's voluntary exhaustion (23). For measurement of respiratory and metabolic variables, a Cortex Metalyzer 3B gas analyzer was used to collect and analyze expired gases. During the test, heart rate (12-lead ECG), blood pressure, and peripheral oxygen saturation were continuously monitored (24). Maximal oxygen consumption (VO₂max) was defined as the highest value achieved during exercise, expressed in absolute terms (L/min) and relative to body mass (mL/kg/min). Test termination criteria followed international guidelines (25): physical exhaustion, athlete's request, limiting symptoms (chest pain, dyspnea incompatible with activity), hemodynamic instability, desaturation (<92%), ST-segment elevation, sustained tachyarrhythmias, and atrioventricular block.

## 3 Results

The descriptive analysis (Table 1) showed that VO₂max values were higher in the U20 category (52.81 ± 5.12 mL·kg^−1^·min^−1^) compared to adults (47.94 ± 5.83 mL·kg^−1^·min^−1^), while U18 athletes presented intermediate values (51.45 ± 3.79 mL·kg^−1^·min^−1^). The right ventricular end-diastolic diameter was greater in U18 athletes (51.50 ± 3.35 mm) compared to U20 (40.56 ± 5.82 mm) and adults (37.22 ± 5.43 mm). The cardiac mass index showed a trend toward progressive increase across categories, although without statistically significant differences. In contrast, left atrial and ventricular dimensions demonstrated an increase in atrial volumes (absolute and indexed) between U20 and adults, whereas left ventricular values remained similar among groups.

**Table 1:** Descriptive statistics of physiological and anatomical indicators by athlete category.

Variable	Category	N	Mean	SD
VO₂max (mL·kg^−1^·min^−1^)	U18	24	51,45	3,789
	U20	16	52,81	5,115
	Adult	18	47,94	5,826
	Total	58	50,74	5,166
Right Ventricular End-Diastolic Diameter (mm)	U18	24	51,50	3,349
	U20	16	40,56	5,819
	Adult	18	37,22	5,429
	Total	58	44,05	7,983
Cardiac Mass Index (g/m²)	U18	24	75,1708	10,76482
	U20	16	78,1688	15,40146
	Adult	18	82,2778	15,09112
	Total	58	78,2034	13,64622
Inferolateral Left Ventricular Wall (mm)	U18	24	7,75	,847
	U20	16	8,38	,806
	Adult	18	8,50	,857
	Total	58	8,16	,894
Interventricular Septum (mm)	U18	24	7,71	,751
	U20	16	8,31	,873
	Adult	18	8,39	1,092
	Total	58	8,09	,942
Ejection Fraction (%) (Teichholz Method)	U18	24	,6579	,04344
	U20	16	,6331	,03790
	Adult	18	,6500	,03804
	Total	58	,6486	,04093
Left Atrial Anteroposterior Diameter (mm)	U18	24	33,67	3,583
	U20	15	32,33	3,039
	Adult	18	31,72	5,004
	Total	57	32,70	3,991
Indexed Left Atrial Anteroposterior Diameter (mm/m^2^)	U18	24	28,0958	2,03202
	U20	15	27,1600	2,07289
	Adult	18	27,4056	2,11005
	Total	57	27,6316	2,07125
Left Atrial Volume (mL)	U18	18	23,57	5,530
	U20	15	38,87	19,935
	Adult	17	51,00	12,629
	Total	50	37,49	17,644
Left Atrial Volume (mL/m^2^)	U18	19	12,20	3,997
	U20	15	20,69	10,215
	Adult	17	26,76	6,068
	Total	51	19,55	9,240
Left Ventricular End-Diastolic Diameter (mm)	U18	24	51,50	3,349
	U20	15	50,20	3,986
	Adult	18	52,00	4,102
	Total	57	51,32	3,766
Indexed Left Ventricular End-Diastolic Diameter (mm/m^2^)	U18	24	28,10	2,032
	U20	15	27,16	2,073
	Adult	18	27,41	2,110
	Total	57	27,63	2,071
Left Ventricular Mass (g)	U18	24	138,4558	24,15158
	U20	15	147,7180	29,48924
	Adult	18	156,7717	32,47609
	Total	57	146,6772	28,96824
Indexed Right Ventricular End-Diastolic Diameter (mm/m²)	U18	24	11,4917	1,93883
	U20	16	14,7250	6,55840
	Adult	18	18,7056	2,67790
	Total	58	14,6224	4,93655

N, subjects; SD, standard deviation.

ANOVA (Table 2) confirmed significant differences between categories for VO_2_max (*p* = 0.013), indexed right ventricular end-diastolic diameter (*p* < 0.001), left ventricular inferolateral wall thickness (*p* = 0.011), interventricular septum thickness (*p* = 0.033), as well as absolute (*p* < 0.001) and indexed (*p* < 0.001) left atrial volume. No differences were observed between groups for ejection fraction, cardiac mass index, left ventricular mass, or left ventricular end-diastolic diameter.

**Table 2:** ANOVA of physiological and anatomical indicators by athlete category.

Variable	Source of variation	DF	Sum of squares	F	*p*
VO₂max (mL·kg^−1^·min^−1^)	Between Groups	2	221,509	4,687	,013
	Within Groups	55	1299,581		
	Total	57	1521,090		
Right Ventricular End-Diastolic Diameter (mm)	Between Groups	2	2365,796	51,347	,000
	Within Groups	55	1267,049		
	Total	57	3632,845		
Cardiac Mass Index (g/m^2^)	Between Groups	2	519,544	1,415	,252
	Within Groups	55	10094,955		
	Total	57	10614,499		
Inferolateral Left Ventricular Wall (mm)	Between Groups	2	6,853	4,864	,011
	Within Groups	55	38,750		
	Total	57	45,603		
Interventricular Septum (mm)	Between Groups	2	5,895	3,629	,033
	Within Groups	55	44,674		
	Total	57	50,569		
Ejection Fraction (%) (Teichholz Method)	Between Groups	2	,006	1,827	,170
	Within Groups	55	,090		
	Total	57	,095		
Left Atrial Anteroposterior Diameter (mm)	Between Groups	2	41,652	1,323	,275
	Within Groups	54	850,278		
	Total	56	891,930		
Indexed Left Atrial Anteroposterior Diameter (mm/m^2^)	Between Groups	2	9,428	1,103	,339
	Within Groups	54	230,815		
	Total	56	240,243		
Left Atrial Volume (mL)	Between Groups	2	6617,951	18,009	,000
	Within Groups	47	8635,549		
	Total	49	15253,500		
Left Atrial Volume Index (mL/m^2^)	Between Groups	2	1931,801	19,835	,000
	Within Groups	48	2337,440		
	Total	50	4269,241		
Left Ventricular End-Diastolic Diameter (mm)	Between Groups	2	27,916	,983	,381
	Within Groups	54	766,400		
	Total	56	794,316		
Indexed Left Ventricular End-Diastolic Diameter (mm/m^2^)	Between Groups	2	9,428	1,103	,339
	Within Groups	54	230,815		
	Total	56	240,243		
Left Ventricular Mass (g)	Between Groups	2	3472,598	2,154	,126
	Within Groups	54	43520,317		
	Total	56	46992,916		
Indexed Right Ventricular End-Diastolic Diameter (mm/m^2^)	Between Groups	2	535,503	17,253	,000
	Within Groups	55	853,558		
	Total	57	1389,061		

DF, degrees of freedom; F, F-statistic; p, statistical significance.

Tukey's multiple comparisons (Table 3) identified the groups responsible for the differences. VO₂max was significantly higher in U20 athletes compared to adults (*p* = 0.014). Indexed right ventricular end-diastolic diameter was greater in U18 athletes than in U20 and adults (*p* < 0.001 for both comparisons). Left ventricular inferolateral wall thickness (*p* = 0.016) and interventricular septum thickness (*p* = 0.048) were greater in adults compared to U18. Left atrial volume, both absolute and indexed, increased progressively from U18 to U20 (*p* = 0.006 and *p* = 0.003, respectively) and from U20 to adults (*p* = 0.039 and *p* = 0.046), with even more pronounced differences between adults and U18 (*p* < 0.001 for both variables). Finally, indexed right ventricular end-diastolic diameter was significantly higher in adults compared to U18 (*p* < 0.001) and U20 (*p* = 0.013), with a difference also observed between U20 and U18 (*p* = 0.036).

**Table 3:** Multiple comparisons (tukey HSD) and significant differences between categories.

Dependent variable	(I) category	(J) category	MD (I-J)	SE	*p*	95% CI	
						Lower bound	Upper bound
VO_2_max (mL·kg^−1^·min^−1^)	U18	U20	−1,362	1,569	,662	−5,14	2,42
		Adult	3,506	1,516	,062	−,14	7,16
	U20	U18	1,362	1,569	,662	−2,42	5,14
		Adult	4,868*	1,670	,014	,85	8,89
	Adult	U18	−3,506	1,516	,062	−7,16	,14
		U20	−4,868*	1,670	,014	−8,89	−,85
Right Ventricular End-Diastolic Diameter (mm)	U18	U20	10,938*	1,549	,000	7,21	14,67
		Adult	14,278*	1,497	,000	10,67	17,88
	U20	U18	−10,938*	1,549	,000	−14,67	−7,21
		Adult	3,340	1,649	,116	−,63	7,31
	Adult	U18	−14,278*	1,497	,000	−17,88	−10,67
		U20	−3,340	1,649	,116	−7,31	,63
Cardiac Mass Index (g/m²)	U18	U20	−2,99792	4,37255	,773	−13,5303	7,5345
		Adult	−7,10694	4,22429	,221	−17,2822	3,0683
	U20	U18	2,99792	4,37255	,773	−7,5345	13,5303
		Adult	−4,10903	4,65494	,653	−15,3216	7,1036
	Adult	U18	7,10694	4,22429	,221	−3,0683	17,2822
		U20	4,10903	4,65494	,653	−7,1036	15,3216
Inferolateral Left Ventricular Wall (mm)	U18	U20	−,625	,271	,063	−1,28	,03
		Adult	−,750*	,262	,016	−1,38	−,12
	U20	U18	,625	,271	,063	−,03	1,28
		Adult	−,125	,288	,902	−,82	,57
	Adult	U18	,750*	,262	,016	,12	1,38
		U20	,125	,288	,902	−,57	,82
Interventricular Septum (mm)	U18	U20	−,604	,291	,104	−1,30	,10
		Adult	−,681*	,281	,048	−1,36	,00
	U20	U18	,604	,291	,104	−,10	1,30
		Adult	−,076	,310	,967	−,82	,67
	Adult	U18	,681*	,281	,048	,00	1,36
		U20	,076	,310	,967	−,67	,82
Ejection Fraction (%) (Teichholz Method)	U18	U20	,02479	,01302	,147	−,0066	,0562
		Adult	,00792	,01258	,805	−,0224	,0382
	U20	U18	−,02479	,01302	,147	−,0562	,0066
		Adult	−,01687	,01386	,448	−,0503	,0165
	Adult	U18	−,00792	,01258	,805	−,0382	,0224
		U20	,01687	,01386	,448	−,0165	,0503
Left Atrial Anteroposterior Diameter (mm)	U18	U20	1,333*	1,306	,567	−1,81	4,48
		Adult	1,944	1,237	,267	−1,04	4,93
	U20	U18	−1,333*	1,306	,567	−4,48	1,81
		Adult	,611*	1,387	,899	−2,73	3,95
	Adult	U18	−1,944	1,237	,267	−4,93	1,04
		U20	−,611*	1,387	,899	−3,95	2,73
Indexed Left Atrial Anteroposterior Diameter (mm/m²)	U18	U20	,93583	,68048	,361	−,7041	2,5758
		Adult	,69028	,64464	,536	−,8633	2,2439
	U20	U18	−,93583	,68048	,361	−2,5758	,7041
		Adult	−,24556	,72279	,938	−1,9875	1,4963
	Adult	U18	−,69028	,64464	,536	−2,2439	,8633
		U20	,24556	,72279	,938	−1,4963	1,9875
Left Atrial Volume (mL)	U18	U20	−15,294*	4,739	,006	−26,76	−3,83
		Adult	−27,428*	4,584	,000	−38,52	−16,33
	U20	U18	15,294*	4,739	,006	3,83	26,76
		Adult	−12,133*	4,802	,039	−23,75	−,51
	Adult	U18	27,428*	4,584	,000	16,33	38,52
		U20	12,133*	4,802	,039	,51	23,75
Left Atrial Volume Index (mL/m²)	U18	U20	−8,496*	2,410	,003	−14,33	−2,67
		Adult	−14,568*	2,330	,000	−20,20	−8,93
	U20	U18	8,496*	2,410	,003	2,67	14,33
		Adult	−6,071*	2,472	,046	−12,05	−,09
	Adult	U18	14,568*	2,330	,000	8,93	20,20
		U20	6,071*	2,472	,046	,09	12,05
Left Ventricular End-Diastolic Diameter (mm)	U18	U20	1,300	1,240	,550	−1,69	4,29
		Adult	−,500	1,175	,905	−3,33	2,33
	U20	U18	−1,300	1,240	,550	−4,29	1,69
		Adult	−1,800	1,317	,365	−4,97	1,37
	Adult	U18	,500	1,175	,905	−2,33	3,33
		U20	1,800	1,317	,365	−1,37	4,97
Indexed Left Ventricular End-Diastolic Diameter (mm/m²)	U18	U20	,936	,680	,361	−,70	2,58
		Adult	,690	,645	,536	−,86	2,24
	U20	U18	−,936	,680	,361	−2,58	,70
		Adult	−,246	,723	,938	−1,99	1,50
	Adult	U18	−,690	,645	,536	−2,24	,86
		U20	,246	,723	,938	−1,50	1,99
Left Ventricular Mass (g)	U18	U20	−9,26217	9,34394	,586	−31,7809	13,2566
		Adult	−18,31583	8,85181	,106	−39,6485	3,0169
	U20	U18	9,26217	9,34394	,586	−13,2566	31,7809
		Adult	−9,05367	9,92486	,635	−32,9724	14,8651
	Adult	U18	18,31583	8,85181	,106	−3,0169	39,6485
		U20	9,05367	9,92486	,635	−14,8651	32,9724
Indexed Right Ventricular End-Diastolic Diameter (mm/m²)	U18	U20	−3,23333*	1,27145	,036	−6,2959	−,1707
		Adult	−7,21389*	1,22834	,000	−10,1726	−4,2551
	U20	U18	3,23333*	1,27145	,036	,1707	6,2959
		Adult	−3,98056*	1,35356	,013	−7,2410	−,7202
	Adult	U18	7,21389*	1,22834	,000	4,2551	10,1726
		U20	3,98056*	1,35356	,013	,7202	7,2410

I, initial comparison category; J, secondary comparison category; MD, mean difference; SE, standard error; p, statistical significance; CI, Confidence interval.

* The mean difference is significant at the 0.05 level.

Overall, the results indicate that, although VO₂max is higher in U20 athletes, adults exhibit greater cardiac remodeling, evidenced by increased ventricular wall thickness, atrial volumes, and right ventricular dimensions when indexed to body surface area.

**Figure 1:**
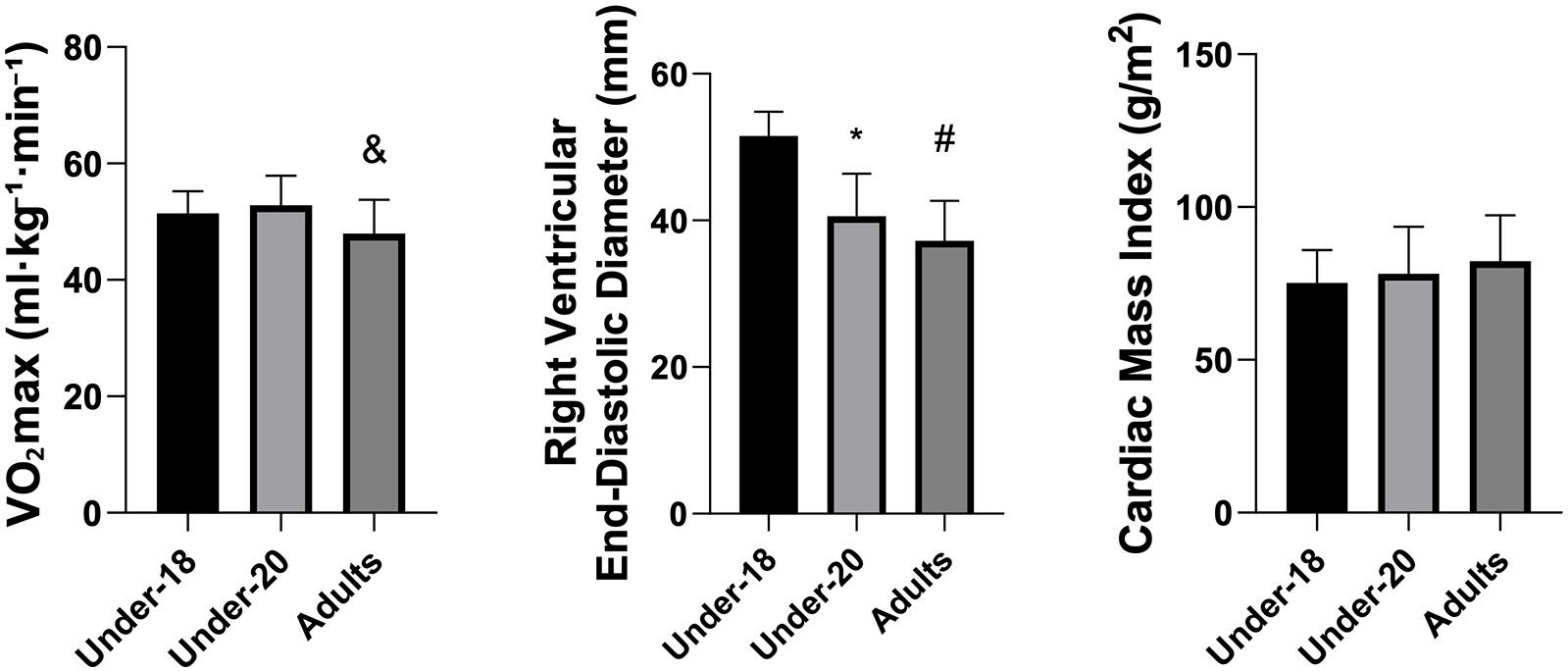
Comparison of VO_2_max (mL·kg^−1^·min^−1^), indexed right ventricular end-diastolic diameter (mm), and cardiac mass Index (g/m^2^) among the under-18, under-20, and adult categories. *Indicates a difference between under-18 and under-20, # indicates a difference between under-18 and adult, and & indicates a difference between under-20 and Adult.

**Figure 2:**
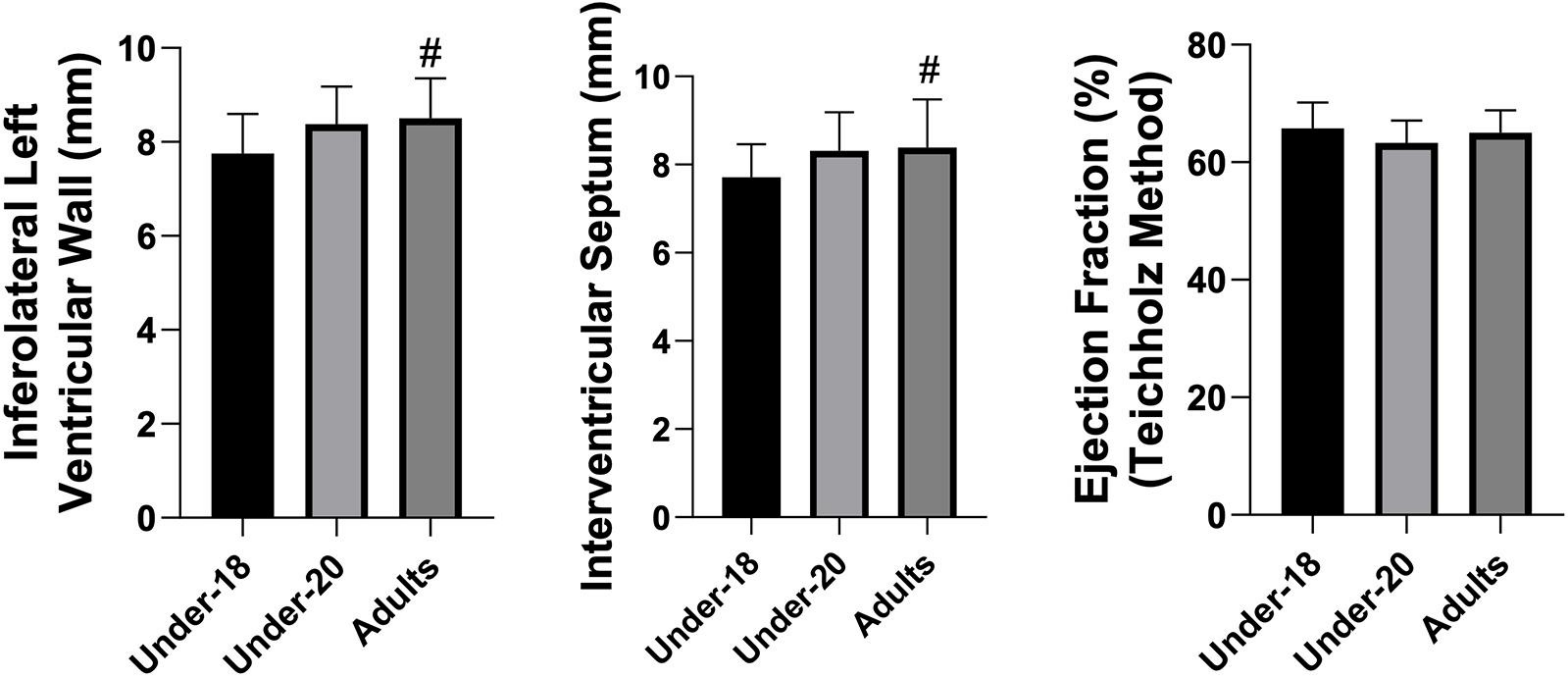
Comparison of inferolateral left ventricular wall (mm), interventricular septum (mm), and ejection fraction (%) (teichholz method) among the under-18, under-20, and adult categories. *Indicates a difference between under-18 and under-20, # indicates a difference between under-18 and adult, and & indicates a difference between under-20 and adult.

**Figure 3:**
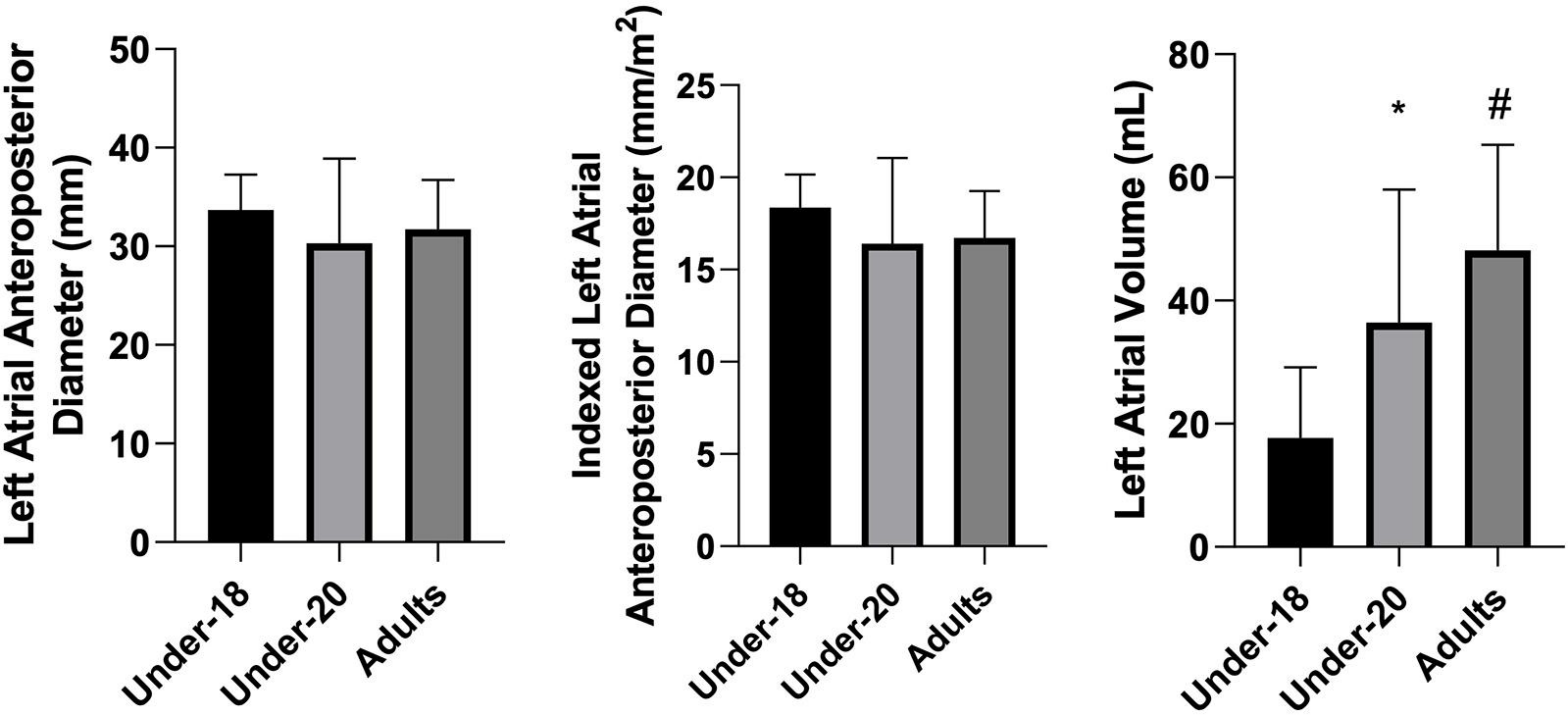
Comparison of left atrial anteroposterior diameter (mm), indexed left atrial anteroposterior diameter (mm/m^2^), and left atrial volume (mL) among the under-18, under-20, and adult categories. *Indicates a difference between under-18 and under-20, # indicates a difference between under-18 and adult, and & indicates a difference between under-20 and adult.

**Figure 4:**
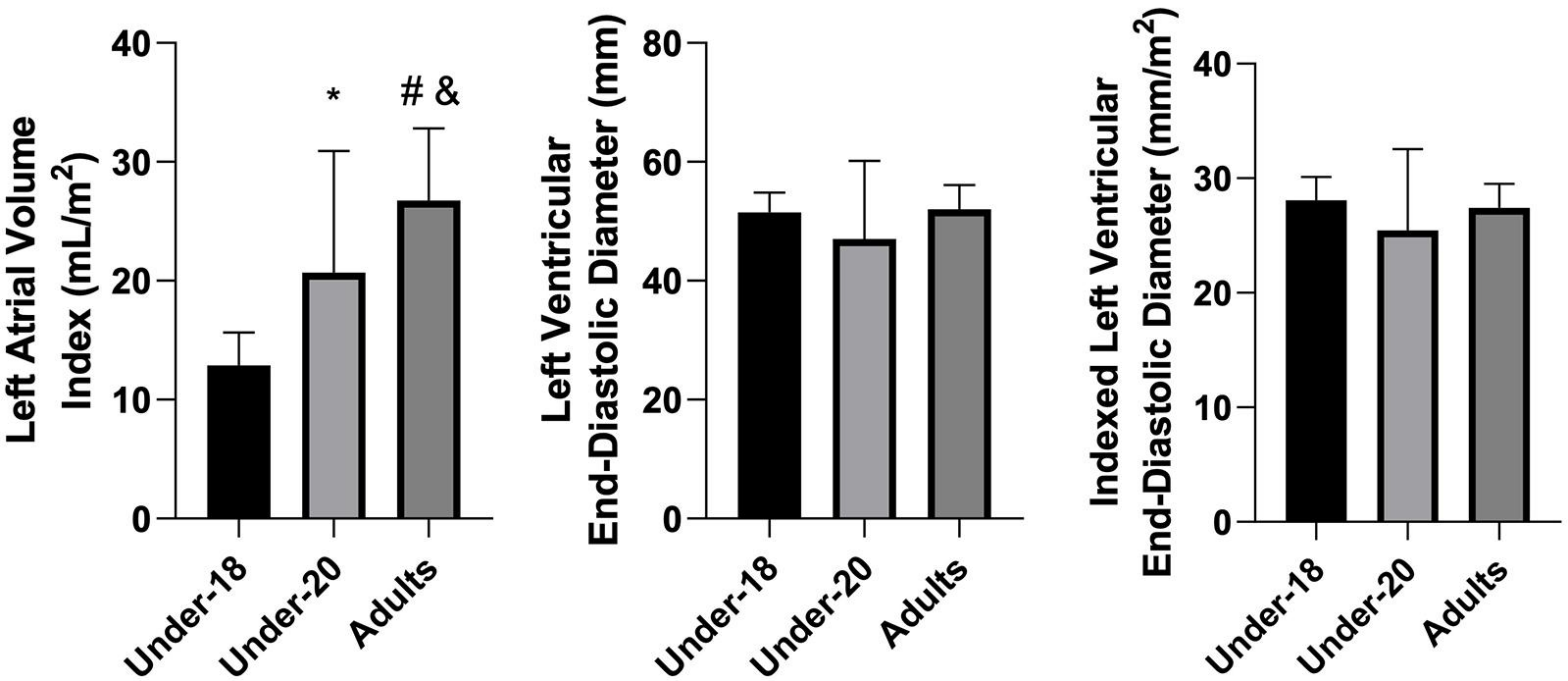
Comparison of left atrial volume Index (mL/m^2^), left ventricular end-diastolic diameter (mm), and indexed left ventricular end-diastolic diameter (mm/m^2^) among the under-18, under-20, and adult categories. *Indicates difference between under-18 and under-20, # indicates difference between under-18 and adult, and & indicates difference between under-20 and adult.

**Figure 5:**
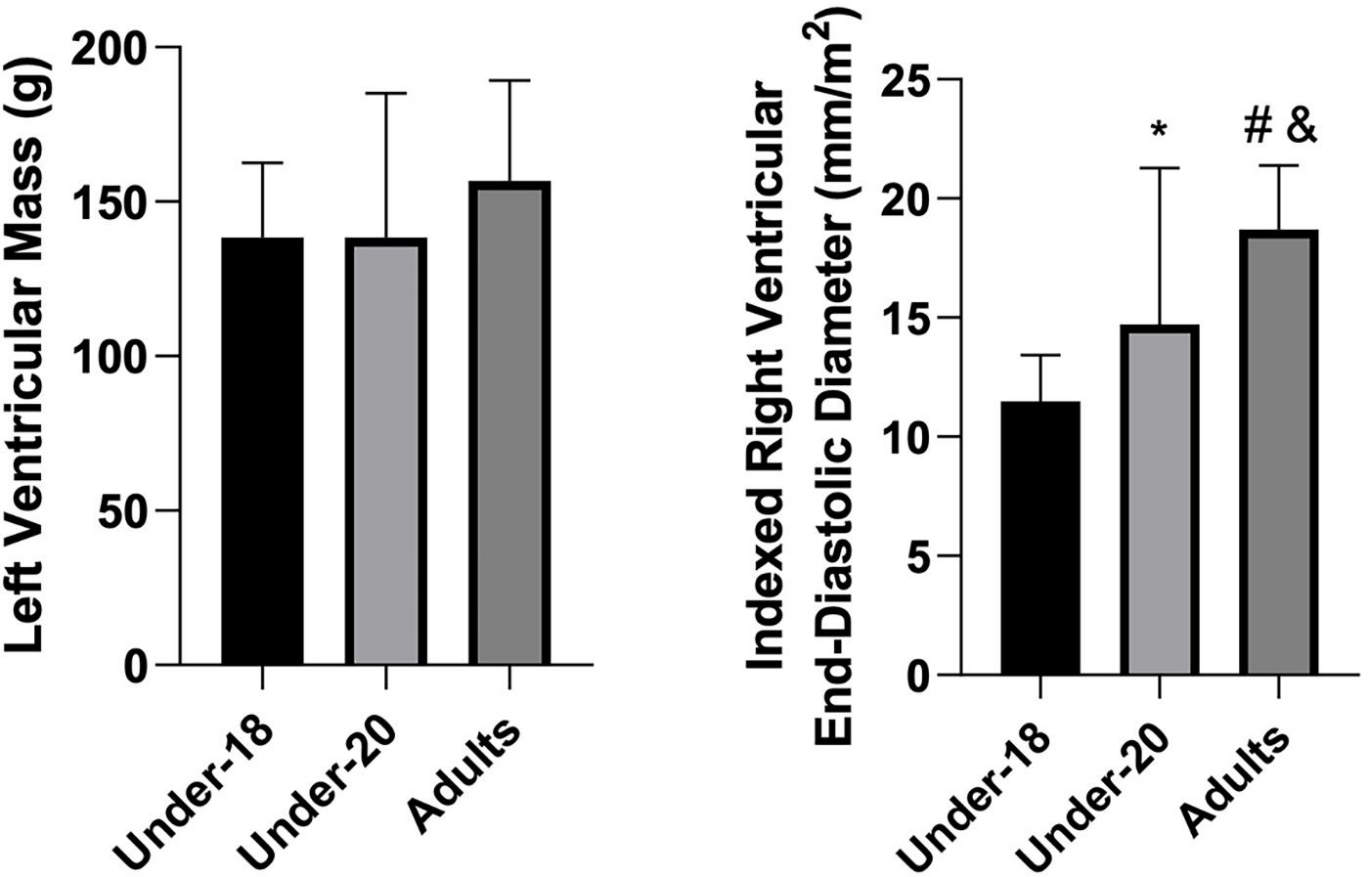
Comparison of left ventricular mass (g) and indexed right ventricular end-diastolic diameter (mm/m^2^) among the under-18, under-20, and adult categories. *Indicates a difference between under-18 and under-20, # indicates a difference between under-18 and adult, and & indicates a difference between under-20 and adult.

Analysis of physiological and anatomical indicators (Figures 1–5) revealed significant differences between categories. VO₂max was higher in the U20 group compared to adults (*p* < 0.05). Regarding cardiac dimensions, absolute and indexed right ventricular end-diastolic diameter was significantly lower in U18 athletes compared to U20 and adults (*p* < 0.05), with an additional difference between U20 and adults in the right ventricular index (*p* < 0.05). Absolute and indexed left atrial volumes also differed, being lower in the U18 group compared to U20 and adults, with an additional difference between U20 and adults for the indexed volume (*p* < 0.05). Furthermore, left ventricular inferolateral wall thickness and interventricular septum thickness were greater in adults compared to the U18 group (*p* < 0.05). In contrast, variables such as cardiac mass index, ejection fraction, left atrial anteroposterior diameter (absolute and indexed), as well as left ventricular diameter and mass, did not show significant differences between categories.

## 4 Discussion

The results of the present study showed that U20 athletes exhibited higher maximal oxygen consumption (VO₂max) compared to adult athletes. This finding may be partially associated with the progressive decline in aerobic capacity that occurs from the third decade of life, shortly after reaching its physiological peak. Although the mean age of the adult category investigated was approximately 24 years, the high age variability observed (standard deviation of 8.8 years) indicates the presence of athletes within age ranges where a reduction in cardiorespiratory fitness is already expected. Moreover, the negative association identified between age and VO₂max supports this hypothesis. Similar results were reported by Parpa et al. (26), who analyzed soccer players and demonstrated that athletes aged 18–24 years had significantly higher VO₂max values compared to athletes aged 25–35 years. In addition, U20 athletes may perform a greater volume of high-intensity actions due to the technical-tactical demands specific to this category. In contrast, more experienced athletes tend to adopt game strategies more focused on tactical control and recovery during training sessions, which may contribute to the differences in VO₂max observed in this study (27). In contrast, more experienced athletes tend to adopt game strategies more focused on tactical control and recovery during training sessions, which may contribute to the differences in VO_2_max observed in this study (7, 27). In fact, the professional athletes evaluated in the present study had a longer cumulative training history and greater competitive experience compared to players from the younger categories, which likely influenced the physiological and tactical distinctions observed between groups. It should be noted, however, that these hypotheses are mostly derived from studies conducted in soccer, and confirmation is needed in future investigations specifically in futsal, given the scarcity of evidence for this sport.

Additionally, Barbero-Álvarez et al. (28) evaluated the physiological demands of ten professional male futsal athletes during four official matches, using heart rate as the primary monitoring variable. The results indicated high aerobic demand, with a mean heart rate of 174 ± 7 bpm, corresponding to 90 ± 2% of HRmax (range 86%–93%). Supporting these findings (17, 18), in a study with simulated matches, demonstrated that athletes spend approximately 46% of the time at intensities above 80% of VO₂max, with an average oxygen consumption around 76% and peaks close to 99% of VO₂max. Complementarily, Bekris et al. (29) observed substantially elevated blood lactate concentrations, reaching 14.9 ± 4.9 mmol/L in the first half and 15.0 ± 4.7 mmol/L in the second half. Collectively, these findings reinforce the characterization of futsal as a high-intensity intermittent sport, marked by successive accelerations, decelerations, and sprints, with or without changes of direction, interspersed with short recovery periods (30).

It can be stated that chronic exposure of athletes to training loads, particularly those related to endurance training, promotes the development of structural and morphological adaptations that make the body more efficient in energy supply during physical effort (31). In this context, the myocardium is expected to exhibit functional, structural, and regulatory adaptations, including increased ventricular mass, changes in cardiac chamber dimensions, and modifications in wall thickness (1, 32, 33).

In the present study, it was observed that more experienced athletes, and therefore with greater accumulated training time, exhibited increased interventricular septum thickness and left ventricular inferior lateral wall thickness. This adaptation is frequently described in the literature among healthy athletes (34). Accordingly, Venckunas et al. (35), when analyzing echocardiographic parameters across different sports modalities, observed that sedentary men had lower measurements compared to all athlete groups. However, basketball and strength/power athletes exhibited greater interventricular septum thickness compared to middle-distance runners. Similarly, Pluim et al. (36) reported that strength and power athletes had higher interventricular septum thickness values (10.5 mm) compared to endurance athletes (8.8 mm), a difference not observed for posterior wall thickness. These data help explain why, the longer the exposure to futsal training a sport characterized by intermittent and high-intensity efforts, in which strength and power are inherently involved in the tactical context of the game the greater the hypertrophic adaptations of the left ventricular inferior lateral wall and interventricular septum. This phenomenon, already documented in previous studies, contributes to understanding why adult futsal athletes exhibit higher values than younger categories.

When analyzing absolute and indexed left atrial volume, ANOVA confirmed significant differences between groups (*p* < 0.001). Tukey's *post-hoc* test revealed the following: U18 < U20, U18 < Adult, and U20 < Adult, for both absolute and indexed volume. These results suggest that, similarly to the hypertrophic adaptations of the left ventricle, progressive exposure to training loads promotes atrial remodeling, resulting in increased left atrial size through a process of eccentric hypertrophy, classically associated with the concept of the “athlete's heart”. Previous studies consistently demonstrate that both training volume and intensity correlate with increased atrial volume (37, 38).

Accordingly, Nistri et al. (39), when evaluating 418 healthy individuals aged 16 years or older, observed a higher indexed left atrial volume in athletes (38.9 ± 9.6 mL/m^2^) compared to non-athletes (28.4 ± 5.8 mL/m^2^). Similarly, D'Andrea et al. (37) reported a high prevalence of mild to moderate left atrial enlargement (27.5%) in a cohort of 615 elite athletes (mean age 28.4 ± 10.2 years; range 18–40), evaluated at a sports medicine reference center.

As previously discussed, in addition to absolute volume, indexed left atrial volume was higher in adult athletes, reflecting not only body size but primarily a chronic cardiac adaptation resulting from systematic training. It is important to emphasize, however, that despite the significant increase in atrial cavity size due to continuous exposure to elevated preload characteristic of the training program the observed dimensions remained within anatomical limits compatible with normal physiological cardiac function, thus representing a benign and adaptive remodeling (40).

Contrary to what might be expected, indexed right ventricular end-diastolic diameter was significantly greater in the U18 group compared to U20 and adults, which did not differ from each other. During intense exercise, this chamber is subjected to disproportionately increased pressures and volumes relative to the left ventricle, due to its smaller muscular mass (approximately one-quarter less than that of the left ventricle) and thinner wall. These factors result in a higher relative workload and greater wall stress in the right ventricle compared to the left. Repetitive volumetric overload, in turn, induces eccentric remodeling characterized by cavity dilation and increased compliance, allowing greater capacity to accommodate blood flow (41).

Our findings are in line with those reported by Augustine et al. (42), who also observed that a considerable proportion of adolescent footballers present right-ventricular dimensions exceeding adult reference ranges. However, while their work established normative values, it did not provide a detailed physiological explanation for this pattern. In our interpretation, the larger right-ventricular diameters in younger athletes may reflect developmental and hemodynamic factors related to cardiac maturation, higher chamber compliance, and predominance of volume-loading stimuli during early athletic training phases.

Furthermore, the progressive deterioration of diastolic function with aging, even in healthy and well-trained individuals, may partially account for the smaller right-ventricular diameters observed in older athletes. Age-related myocardial changes, including increased peripheral resistance, extracellular matrix proliferation, collagen cross-linking, and altered calcium handling, are known to reduce ventricular compliance and impair relaxation (43). Aging alone, however, may not totally explain all the changes that occur during myocardial senescence. Other factors that change with age such as diminishing cardiovascular fitness could also contribute to the gradual decrease of diastolic function. Thus, the greater right-ventricular dimensions observed in U18 athletes likely reflect not only the adaptive response to exercise stimuli but also the inherent physiological advantage of a younger, more compliant myocardium.

On the other hand, several analyzed parameters did not show significant differences between groups, including left atrial anteroposterior diameter, ejection fraction, left ventricular end-diastolic diameter (absolute and indexed), and left ventricular mass index. Specifically regarding the left atrium, although an increase in volume was observed, anteroposterior diameter did not follow the same trend. This result may be explained by atrial chamber geometry, which does not expand uniformly in all directions (44). In other words, atrial volume can increase substantially without significant changes in the anteroposterior dimension, which is a unidimensional measure and may not accurately reflect the true size of the chamber. For this reason, current guidelines recommend assessing the left atrium via volumetric measurement (biplane method) rather than linear anteroposterior diameter (20). The findings of the present study support this recommendation: the absence of differences in anteroposterior diameter, contrasted with clear volumetric increases, indicates that only volumetric assessment was able to capture the atrial remodeling effectively present in older athletes.

Left ventricular ejection fraction was similar across the three groups, with no statistically significant differences, and all values remained within normal limits. This result was expected, as in the athlete's heart, systolic function tends to remain normal despite changes in cardiac geometry. Supporting these findings, Cabanelas, Freitas, and Gonçalves (45) analyzed soccer athletes using echocardiography and observed that ejection fraction did not vary significantly in serial evaluations, indicating preserved contractile function under basal conditions. Similarly, our results suggest that increases in atrial volume and other structural adaptations did not compromise systolic performance, reinforcing that this represents a physiological adaptive process. This pattern contrasts with pathological conditions, where morphological and structural chamber alterations are generally associated with functional impairment. Preservation of left ventricular ejection fraction around 60%, or slightly lower values in endurance athletes at rest, can be interpreted as part of a functional adjustment: larger cardiac chambers can maintain efficient output through compensatory mechanisms (Frank-Starling law) and increased resting vagal tone, which may slightly reduce ejection fraction without clinical implications.

Left ventricular end-diastolic diameter, both absolute and indexed, did not differ significantly between groups. This suggests that left ventricular dimensions may already be near a plateau of adaptation to training from youth. Additionally, from the age of 18, the heart may preferentially adapt through wall hypertrophy rather than further internal diameter expansion concentric vs. eccentric hypertrophy (45). Increases in left ventricular end-diastolic diameter are classically associated with dynamic sports involving high exercise volumes, which induce greater cardiac output and blood pressure load. This characteristic, however, differs partially from futsal, a mixed sport in which intermittent demands for strength, power, and endurance coexist (30, 36). These hypotheses also help explain the absence of increased left ventricular mass index from U18 to professional levels, as observed in our findings.

Finally, although some structural adaptations observed in this study reinforce a chronic training-induced adaptive process, it should be considered that futsal, compared to classical endurance sports, retains hybrid characteristics, combining strength and power elements with substantial aerobic demands. This mixed nature may result in morphological and structural adaptations that are less pronounced than those observed in predominantly aerobic sports, such as long-distance running (46–48). For comparison, Sjödin and Svedenhag (49) reported mean VO₂max values of 71.8 ± 1.2 mL/kg/min in long-distance runners, whereas the mean obtained in the sample of the present study was 50.7 ± 5.17 mL/kg/min approximately 30% lower. This difference may be directly related to the absence of more pronounced chronic adaptations in certain structural variables in the futsal athlete group evaluated.

## 5 Conclusion

The present study identified significant differences in cardiorespiratory and cardiac structural variables among under-18, under-20, and adult futsal athletes, particularly in VO₂max, right ventricular end-diastolic diameter, ventricular wall thickness, and left atrial volume indices. Adult athletes exhibited higher values in several parameters, reflecting cumulative physiological adaptations associated with increased competitive demands and prolonged training exposure. These results support the relationship between sport progression and cardiac remodeling characteristic of the “athlete's heart”, with implications for clinical assessment and monitoring of athletes transitioning to higher performance levels.

Practically, the findings may assist clinicians and coaches in interpreting cardiac adaptations across developmental stages, aiding in the identification of expected physiological responses and reducing the risk of misinterpreting adaptive changes as pathological. They also contribute to individualized monitoring strategies aimed at ensuring safety and optimizing performance.

This study has limitations. Its cross-sectional design prevents causal inferences regarding training duration, competitive level, and cardiac remodeling; longitudinal research is needed to better characterize cardiovascular adaptation throughout athletes' careers. Additionally, previous training load data could not be standardized due to player transfers, and data collection occurred before the start of the season, as cardiological evaluations are required prior to organized training.

Finally, although current echocardiographic guidelines recommend 2D linear measurements for chamber quantification, M-mode was used when optimal acoustic windows were available, and volumetric measures were obtained from 2D images. This methodological choice, consistent with clinical practice, should be acknowledged when interpreting the results.

## 6 Limitations

This study presents as a limitation its cross-sectional design, which may hinder the establishment of causal relationships between age, training exposure, and cardiac or cardiorespiratory adaptations. Therefore, longitudinal studies are necessary to confirm these findings. Previous training load could not be standardized due to athlete transfers, and assessments were conducted before the beginning of the competitive season, which limits control over recent training stimuli.

Another limitation concerns the indexation of cardiac parameters exclusively to body surface area, although widely recommended (20); alternative indexation methods may provide additional insights. Moreover, although extracurricular physical activity during the season is contraindicated for professional athletes, information on previous-season activity outside current club environments was unavailable. Still, the short off-season typical of futsal is unlikely to induce substantial chronic adaptations.

Additionally, some interpretations are based on evidence from soccer because of the scarcity of futsal-specific studies, which may restrict the precision of modality-specific inferences. Finally, maturational factors in younger athletes may have contributed to the differences observed in right ventricular end-diastolic diameter, and the mixed physiological nature of futsal may partially explain the absence of more pronounced cardiac adaptations in some variables.

## Data Availability

The original contributions presented in the study are included in the article/Supplementary Material, further inquiries can be directed to the corresponding author.

## References

[1] Morganroth J Maron BJ Henry W Epstein SE. Comparative left ventricular dimensions in trained athletes. Ann Intern Med. (1975) 82:521–4. 10.7326/0003-4819-82-4-521 1119766

[2] Rawlins J Bhan A Sharma S. Left ventricular hypertrophy in athletes. Eur J Echocardiogr. (2009) 10:350–6. 10.1093/ejechocard/jep017 19246500

[3] Neri V Ambrosi A Fersini A Tartaglia N Lapolla F Forlano I. Severe acute pancreatitis: clinical forms of different gravity. Ann Ital Chir. (2013) 84:47–53. 23449167

[4] Lamotte M Fleury F Pirard M Jamon A van de Borne P. Acute cardiovascular response to resistance training during cardiac rehabilitation: effect of repetition speed and rest periods. Eur J Prev Cardiol. (2010) 17:329–36. 10.1097/HJR.0b013e328332efdd 20104178

[5] Arena R Myers J Guazzi M. The clinical significance of aerobic exercise testing and prescription: from apparently healthy to confirmed cardiovascular disease. Am J of Lifestyle Med. (2008) 2(6):519–36. 10.1177/1559827608323210

[6] Rawlins J Carre F Kervio G Papadakis M Chandra N Edwards C Ethnic differences in physiological cardiac adaptation to intense physical exercise in highly trained female athletes. Circulation. (2010) 121(9):1078–85. 10.1161/CIRCULATIONAHA.109.917211 20176985

[7] Bangsbo J Mohr M Krustrup P. Physical and metabolic demands of training and match-play in the elite football player. J Sports Sci. (2006) 24:665–74. 10.1080/02640410500482529 16766496

[8] Bramanti V Tomassoni D Grasso S Bronzi D Napoli M Campisi A Cholinergic precursors modulate the expression of heme oxigenase-1, p21 during astroglial cell proliferation and differentiation in culture. Neurochem Res. (2012) 37:2795–804. 10.1007/s11064-012-0873-3 22956150

[9] Mielgo-Ayuso J Maroto-Sánchez B Luzardo-Socorro R Palacios G Palacios Gil-Antuñano N González-Gross M. Evaluation of nutritional status and energy expenditure in athletes. Nutr Hosp. (2015) 31:227–36. 10.3305/nh.2015.31.sup3.8770 25719790

[10] Francavilla CV Sessa F Salerno M Albano GD Villano I Messina G Influence of football on physiological cardiac indexes in professional and young athletes. Front Physiol. (2018) 9:153. 10.3389/fphys.2018.00153 29541036 PMC5835836

[11] Vyshka G Vacchiano G. Severe flaccid paraparesis following spinal anaesthesia: a sine materia occurrence. BMJ Case Rep. (2014) 2014:bcr2013202071. 10.1136/bcr-2013-202071 24832705 PMC4024567

[12] Lamotte M Niset G Van de Borne P. The effect of different modalities of resistance training on beat to beat blood pressure in cardiac patients. Eur J Cardiovasc Prev Rehabil. (2005) 12:12–7. 15703501

[13] Rodrigues VM Ramos GP Mendes TT Cabido CE Melo ES Condessa LA Intensity of official futsal matches. J Strength Cond Res. (2011) 25(9):2482–7. 10.1519/JSC.0b013e3181fb4574 21869629

[14] Nunes RFH Almeida FAM Santos BV Almeida FDM Nogas G Elsangedy HM Comparação de indicadores físicos e fisiológicos entre atletas profissionais de futsal e futebol. Motriz: Revista de Educação Física. (2012) 18:104–12. 10.1590/S1980-65742012000100011

[15] Zurutuza U Castellano J Echeazarra I Casamichana D. Absolute and relative training load and its relation to fatigue in football. Front Psychol. (2017) 8:878. 10.3389/fpsyg.2017.00878 28634456 PMC5459919

[16] Barbieri FA Barbieri RA Queiroga MR Santana WC Kokubun E. Perfil antropométrico e fisiológico de atletas de futsal da categoria sub-20 e adulta. Motricidade. (2012) 8(4):62–70. 10.6063/motricidade.8(4).1553

[17] Castagna C Impellizzeri F Cecchini E Rampinini E Álvarez JCB. Effects of intermittent-endurance fitness on match performance in young male soccer players. J Strength Cond Res. (2009) 23(7):1954–9. 10.1519/JSC.0b013e3181b7f743 19855318

[18] Castagna C D’Ottavio S Vera JG Álvarez JCB. Match demands of professional futsal: a case study. J Sci Med Sport. (2009) 12(4):490–4. 10.1016/j.jsams.2008.02.001 18554983

[19] Drezner JA Sharma S Baggish A Papadakis M Wilson MG Prutkin JM International criteria for electrocardiographic interpretation in athletes: consensus statement. Br J Sports Med. (2017) 51(9):704–31. 10.1136/bjsports-2016-097331 28258178

[20] Lang RM Badano L Mor-Avi V Afilalo J Armstrong A Ernande L Recommendations for cardiac chamber quantification by echocardiography in adults: an update from the American society of echocardiography and the European association of cardiovascular imaging. J Am Soc Echocardiogr. (2015) 28(1):1–39.e14. 10.1016/j.echo.2014.10.003 25559473

[21] Teichholz LE Kreulen T Herman MV Gorlin R. Problems in echocardiographic volume determinations: echocardiographic-angiographic correlations in the presence or absence of asynergy. Am J Cardiol. (1976) 37(1):7–11. 10.1016/0002-9149(76)90491-4 1244736

[22] Devereux RB Alonso DR Lutas EM Gottlieb GJ Campo E Sachs I Echocardiographic assessment of left ventricular hypertrophy: comparison to necropsy findings. Am J Cardiol. (1986) 57(6):450–8. 10.1016/0002-9149(86)90771-x 2936235

[23] Guazzi M Arena R Halle M Piepoli MF Myers J Lavie CJ. 2016 Focused update: clinical recommendations for cardiopulmonary exercise testing data assessment in specific patient populations. Eur Heart J. (2018) 39(14):1144–61. 10.1093/eurheartj/ehw180 27141094

[24] Dores H Antunes M Caldeira D Pereira HV. Cardiovascular benefits of resistance exercise: it’s time to prescribe. Rev Port Cardiol. (2024) 43(10):573–82. 10.1016/j.repc.2024.02.009 38703948

[25] American College of Sports Medicine. ACSM’s Guidelines for Exercise Testing and Prescription. 10th ed. Philadelphia: Wolters Kluwer (2021).

[26] Parpa K Michaelides M. Anthropometric characteristics and aerobic performance of professional soccer players by playing position and age. Hum Mov. (2022) 23(4):44–53. 10.5114/hm.2022.110124

[27] Mendez-Villanueva A Buchheit M Simpson B Bourdon PC. Match play intensity distribution in youth soccer. Int J Sports Med. (2013) 34(2):101–10. 10.1055/s-0032-1306323 22960988

[28] Barbero-Álvarez JC Soto VM Barbero-Álvarez V Granda-Vera J. Match analysis and heart rate of futsal players during competition. J Sports Sci. (2008) 26(1):63–73. 10.1080/02640410701287289 17899472

[29] Bekris E Gioldasis A Gissis I Katis A Mitrousis I Mylonis E. Effects of a futsal game on metabolic, hormonal, and muscle damage indicators of male futsal players. J Strength Cond Res. (2022) 36(2):545–50. 10.1519/JSC.0000000000003466 32032230

[30] Caetano FG de Oliveira Bueno MJ Marche AL Nakamura FY Cunha SA Moura FA. Characterization of the sprint and repeated-sprint sequences performed by professional futsal players, according to playing position, during official matches. J Appl Biomech. (2015) 31:423–9. 10.1123/jab.2014-0159 26155741

[31] Buchheit M Laursen PB. High-intensity interval training, solutions to the programming puzzle: part I: cardiopulmonary emphasis. Sports Med. (2013) 43(5):313–38. 10.1007/s40279-013-0029-x 23539308

[32] Maron BJ Levine BD Washington RL Baggish AL Kovacs RJ Maron MS Eligibility and disqualification recommendations for competitive athletes with cardiovascular abnormalities: task force 2: preparticipation screening for cardiovascular disease in competitive athletes: a scientific statement from the American heart association and American college of cardiology. Circulation. (2015) 132:e267–72. 10.1016/j.jacc.2015.09.034 26527714

[33] De Innocentiis C Ricci F Khanji MY Aung N Tana C Verrengia E Athlete’s heart: diagnostic challenges and future perspectives. Sports Med. (2018) 48:2463–77. 10.1007/s40279-018-0985-2 30251086

[34] Boraita A Díaz-Gonzalez L Valenzuela PL Heras ME Morales-Acuna F Castillo-García A Normative values for sport-specific left ventricular dimensions and exercise-induced cardiac remodeling in elite Spanish male and female athletes. Sports Med Open. (2022) 8(1):116. 10.1186/s40798-022-00510-2 36107355 PMC9478009

[35] Venckunas T Semenova EA Zempo H Pranckeviciene E Galkavicius G Stadnik A Echocardiographic parameters in athletes of different sports. J Sports Sci Med. (2008) 7(1):151–6. 24150148 PMC3763341

[36] Pluim BM Zwinderman AH van der Laarse A van der Wall EE. The athlete’s heart: a meta-analysis of cardiac structure and function. Circulation. (2000) 101(3):336–44. 10.1161/01.CIR.101.3.336 10645932

[37] D’Andrea A Riegler L Cocchia R Scarafile R Salerno G Gravino R Left atrial volume index in highly trained athletes. Am Heart J. (2010) 159(6):1155–61. 10.1016/j.ahj.2010.03.036 20569734

[38] Iskandar A Mujtaba MT Thompson PD. Left atrium size in elite athletes. JACC Cardiovasc Imaging. (2015) 8(7):753–62. 10.1016/j.jcmg.2014.12.032 26093921

[39] Nistri S Galderisi M Ballo P Lisi M Santoro C D’Andrea A Determinants of echocardiographic left atrial volume: implications for normalcy. Eur J Echocardiogr. (2011) 12(11):826–33. 10.1093/ejechocard/jer137 21880608

[40] Babio GD Janavel GV Constantin I Masson G Carrero C Botta TG Atrial size and sports. A great training for a greater left atrium: how much is too much? Int J Cardiovasc Imaging. (2021) 37(3):981–8. 10.1007/s10554-020-02082-2 33104945

[41] D’Andrea A Morello A Iacono AM Scarafile R Cocchia R Riegler L Right ventricular changes in highly trained athletes: between physiology and pathophysiology. J Cardiovasc Echogr. (2015) 25(4):97–102. 10.4103/2211-4122.172486 28465945 PMC5353418

[42] Augustine DX Willis J Sivalokanathan S Wild C Sharma A Zaidi A Right ventricular assessment of the adolescent footballer’s heart. Echo Res Pract. (2024) 11(1):7. 10.1186/s44156-023-00039-4 38424646 PMC10905853

[43] Teske AJ Prakken NHJ De Boeck BWL Velthuis BK Martens EP Doevendans PA Effect of long-term and intensive endurance training in athletes on the age-related decline in left and right ventricular diastolic function as assessed by Doppler echocardiography. Am J Cardiol. (2009) 104(8):1145–51. 10.1016/j.amjcard.2009.05.066 19801039

[44] Abhayaratna WP Seward JB Appleton CP Douglas PS Oh JK Tajik AJ Left atrial size: physiologic determinants and clinical applications. J Am Coll Cardiol. (2006) 47(12):2357–63. 10.1016/j.jacc.2006.02.048 16781359

[45] Cabanelas N Freitas S Gonçalves L. Evolução das características morfofuncionais do coração do atleta durante uma época desportiva. Rev Port Cardiol. (2013) 32(4):291–6. 10.1016/j.repc.2012.06.015 23518395

[46] Thompson PD. Cardiovascular adaptations to marathon running: the marathoner’s heart. Sports Med. (2007) 37(4):444–7. 10.2165/00007256-200737040-00045 17465631

[47] Spyrou K Freitas TT Marín-Cascales E Alcaraz PE. Physical and physiological match-play demands and player characteristics in futsal: a systematic review. Front Psychol. (2020) 11:569897. 10.3389/fpsyg.2020.569897 33240157 PMC7677190

[48] Kandels J Metze M Stöbe S Do L Möbius-Winkler MN Antoniadis M Long-term follow-up of professional soccer players: analyses of left and right heart morphology and function by conventional, three-dimensional, and deformation analyses. Diagnostics. (2025) 15(14):1745. 40722494 10.3390/diagnostics15141745 PMC12293576

[49] Sjödin B Svedenhag J. Applied physiology of marathon running. Sports Med. (1985) 2(2):83–99. 10.2165/00007256-198502020-00002 3890068

